# Possible involvement of distinct phylogenetic clusters of HIV-1 variants in the discrepancies between coreceptor tropism predictions based on viral RNA and proviral DNA

**DOI:** 10.1186/s40780-016-0065-4

**Published:** 2016-11-09

**Authors:** Hiroshi Kotani, Koji Sudo, Naoki Hasegawa, Hiroshi Fujiwara, Tomohisa Hayakawa, Osamu Iketani, Masaya Yamaguchi, Mayumi Mochizuki, Satoshi Iwata, Shingo Kato

**Affiliations:** 1 Department of Pharmacy, Keio University Hospital, 35 Shinanomachi, Shinjuku-ku, Tokyo, 160-8582 Japan; 2 Center for Infectious Diseases and Infection Control, Keio University School of Medicine, 35 Shinanomachi, Shinjuku-ku, Tokyo, 160-8582 Japan; 3 Department of Microbiology and Immunology, Keio University School of Medicine, 35 Shinanomachi, Shinjuku-ku, Tokyo, 160-8582 Japan; 4 Faculty of Pharmacy, Keio University, 1-5-30 Shibakoen, Minato-ku, Tokyo, 105-8512 Japan

**Keywords:** CCR5-antagonisit, HIV-1, Coreceptor tropism, viral RNA, Proviral DNA, Deep sequencing

## Abstract

**Background:**

The coreceptor tropism testing should be conducted prior to commencing a regimen containing a CCR5 antagonist for treatment of HIV-1 infection. For aviremic patients on long antiretroviral therapy, proviral DNA is often used instead of viral RNA in genotypic tropism testing. However, the tropism predictions from RNA and DNA are sometimes different. We examined the cause of the discrepancies between HIV-1 tropism predictions based on viral RNA and proviral DNA.

**Methods:**

The nucleotide sequence of the *env* C2V3C3 region was determined using pair samples of plasma RNA and peripheral blood mononuclear cell DNA from 50 HIV-1 subtype B-infected individuals using population-based sequencing. The samples with discrepant tropism assessments between RNA and DNA were further analyzed using deep sequencing, followed by phylogenetic analysis. The tropism was assessed using the algorithm geno2pheno with a false-positive rate cutoff of 10 %.

**Results:**

In population-based sequencing, five of 50 subjects showed discrepant tropism predictions between their RNA and DNA samples: four exhibited R5 tropism in RNA and X4 tropism in DNA, while one exhibited the opposite pattern. In the deep sequencing and phylogenetic analysis, three subjects had single clusters comprising of RNA- and DNA-derived sequences that were a mixture of R5 and X4 sequences. The other two subjects had two and three distinct phylogenetic clusters of sequences, respectively, each of which was dominated by R5 or X4 sequences; sequences of the R5-dominated cluster were mostly found in RNA, while sequences of the X4-dominated cluster were mostly in DNA.

**Conclusions:**

Some of HIV-1 tropism discrepancies between viral RNA and proviral DNA seem to be caused by phylogenetically distinct clusters which resides in plasma and cells in different frequencies. Our findings suggest that the tropism testing using PBMC DNA or deep sequencing may be required when the viral load is not suppressed or rebounds in the course of a CCR5 antagonist-containing regimen.

## Background

HIV-1 enters host cells through the binding of the surface envelope glycoprotein (gp120) to the receptor CD4 and the principle coreceptor CXCR4 or CCR5, which are members of the G protein-coupled family of chemokine receptors. According to coreceptor usage, HIV-1 variants are designated as X4 (CXCR4-specific), R5 (CCR5-specific), or R5X4 (using both CCR5 and CXCR4) [1]. Maraviroc (MVC), which is the only CCR5 antagonist approved by the US Food and Drug Administration, prevents the binding and entry of R5 viruses exclusively. Therefore, a coreceptor tropism assay is strongly recommended whenever the use of a CCR5 antagonist is being considered [2, 3].

Coreceptor usage can be determined using either a phenotypic or a genotypic assay. The most widely used phenotypic assay is the Trofile assay (Monogram Biosciences, South San Francisco, CA), which was used in early clinical trials of MVC [4, 5]. This assay has been improved and is now available as the enhanced-sensitivity Trofile assay [6]. Genotypic assays are based on the sequence of the *env* V3-coding region, which is the principal determinant of co-receptor usage, followed by interpretation using a variety of bioinformatic algorithms. Currently, the most widely interpretation system for tropism is geno2pheno [co-receptor] (G2P), the performance of which is equivalent to that of phenotyping for predicting the therapeutic response to MVC [7, 8], while it has been reported that G2P is not always accurate for non-B subtypes [9]. Although more evidence supports the phenotypic assay, genotypic assays are being increasingly used in clinical settings because of their lower cost, higher throughput, and greater accessibility [3].

Genotypic assays are commonly performed and evaluated using plasma viral RNA in patients when the viral load is high enough for PCR amplification (ideally >1,000 copies/ml). However, for patients whose viral load is suppressed by successful antiretroviral therapy (ART), a genomic assay using viral RNA cannot be performed. Even in such patients, a tropic assay is needed if a change to a MVC-containing regimen is considered because of the adverse effects of or nonadherence to the current regimen. For such patients, tropism testing using proviral DNA in peripheral blood mononuclear cells (PBMCs) is a feasible alternative [3] because proviral DNA decays with a significantly longer half-life than viral RNA during ART [10, 11]. However, the coreceptor usage predicted from proviral DNA is not the same as that from viral RNA in all cases. Studies comparing DNA and RNA tropism assays have reported concordance rates ranging from 78 to 100 % [12–23]. In general, X4-tropic sequences are more frequently drawn from proviral DNA than from viral RNA (clinical meanings) [12, 17, 18, 20, 23], but opposite results have been reported in some studies [16, 19, 21]. However, the causes of these discrepancies are poorly understood.

In this study, we compared the coreceptor tropisms determined by genotypic assays using viral RNA and proviral DNA in 50 treatment-naïve patients and analyzed five paired samples with discrepant tropisms using deep sequencing, followed by a phylogenetic analysis to elucidate the cause of such discrepancies.

## Methods

### Study population

Whole blood samples (anticoagulant, citrate dextrose) were obtained from 50 treatment-naive patients infected with HIV-1 who attended the Infectious Disease Clinic at Keio University Hospital, Tokyo, Japan. This study was approved by the Ethics Committee of Keio University School of Medicine (approval number, 2011–011). Written informed consent was obtained from all the participants.

### Sample preparation

Plasma and PBMCs were separated on a Ficoll-Paque PLUS gradient (GE Healthcare, Tokyo, Japan). RNA was extracted from the plasma using the QIAamp® Viral RNA Mini Kit (Qiagen, Tokyo, Japan), and DNA was extracted from the PBMCs using the QIAamp® DNA Blood Mini Kit (Qiagen) according to the manufacturer’s instructions.

### Quantitation of viral RNA and proviral DNA

Viral RNA load in plasma was determined at a reference laboratory using COBAS Ampliprep/COBAS TaqMan HIV-1 v.2.0 assay (Roche Diagnostic, Basel, Switzerland). Proviral DNA load was determined as previously described [24].

### Drug resistance mutations

Drug resistance mutations were assessed using the HIV-1 drug resistance testing standardized by the Japanese external quality assessment program [25].

### Population-based sequencing

Population-based sequencing was performed as follows. The HIV-1 *env* C2V3C3 region was amplified from 10 μl of plasma RNA solution (corresponding to RNA from 23 μl of plasma) or 250 ng of PBMC DNA with nested PCR using a forward primer (5′-GTCAGCACAGTACAATGYACACATGG-3′, corresponding to nucleotides 6948–6973 of HXB2 [accession number K03455]) and a reverse primer (5′-TGTGTTGTATTACAGTAGAAAAATTCYCC-3′, 7362–7390) for the first-found PCR, and a forward primer (5′-GCTGTTAAATGGCAGTYTAGCAGA-3′, 7001–7024) and a reverse primer (5′-AATTTCTGGGTCYCCTCCTG-3′, 7318–7337) for the second-round PCR. A set of RT and first-round PCR was performed in 25 μl of reaction mixture containing 1× PCR Buffer (attached to Platinum® Taq DNA Polymerase, Life Technologies, Tokyo, Japan), 2 mM MgCl_2_, 0.2 mM of each dNTP, 0.2 mM of forward and reverse primers, 2 units of RNasin® RNase Inhibitor (Promega, Tokyo, Japan), 10 units of SuperScript® III Reverse Transcriptase, and 0.1 μl of Platinum® Taq DNA Polymerase (Life Technologies). Second-round PCR was performed using 1 μl of the first-round PCR product in 25 μl of reaction mixture containing 1× PCR buffer, 2 mM MgCl_2_, 0.2 mM of each dNTP, 0.2 mM of forward and reverse primers, and 0.1 μl of Platinum® Taq DNA Polymerase. The following cycling parameters were used: for RT and first-round PCR, 50 °C for 10 min, 94 °C for 1 min, 5 cycles of 5 s at 94 °C, 10 s at 48 °C and then 30 s at 72 °C, 25 cycles of 5 s at 94 °C, 10 s at 52 °C and then 30 s at 72 °C, followed by 1 min at 72 °C and holding at 4 °C; for second-round PCR, 94 °C for 1 min, 5 cycles of 5 s at 94 °C, 48 °C for 10 s and 72 °C for 30 s, 30 cycles of 5 s at 94 °C, 10 s at 60 °C and then 30 s at 72 °C, followed by 1 min at 72 °C and holding at 4 °C. The PCR products were purified using the QIAquick® PCR Purification Kit (Qiagen) and were subjected to bidirectional population sequencing with the second-round PCR primers and the BigDye® Terminator v1.1 Cycle Sequencing Kit (Life Technologies) in the 3130xL Genetic Analyzer, following the manufacturer’s instructions.

### Subtyping

Subtyping was performed by the phylogenetic analysis using the *env* C2V3C3 sequences of samples and subtype-specific reference variants obtained from the Los Alamos HIV databases (http://www.hiv.lanl.gov/).

### Deep sequencing

For deep sequencing, RT-nested PCR was performed as above except for the use of Platinum® Taq DNA Polymerase High Fidelity instead of Platinum® Taq DNA Polymerase. Deep sequencing was performed using the GS Junior system (Roche Diagnostics, Tokyo, Japan); the second-round PCR products were purified using the QIAquick® PCR Purification Kit (Qiagen). The second-round PCR products were bound with multiplex tags, allowing 12 samples to be sequenced in both the forward and reverse directions per run. A library of the resulting PCR products was produced using the GS Junior Titanium Sequencing Kit. Emulsion PCR was performed with the GS Junior Titanium emPCR Kit. The DNA-carrying beads were deposited into the wells of a PicoTiterPlate (PTP) device, and the nucleotide sequence was determined using the GS Junior Titanium Sequencing Kit.

Nucleotide reads from the system were first sorted using multiplex tags and primers. Sequencing reads with differences in the tag and primer nucleotide sequences were excluded. Sequencing reads in which insertions and deletions (occurring mainly in homopolymeric regions) caused frame-shifts were also excluded. Identical nucleotide reads were merged and defined as a variant; a variant with a frequency of less than 1 % was considered to have been produced by errors during PCR and deep sequencing. This threshold was based on the observation that PCR amplification followed by deep sequencing of three HIV-1 LAI RNA clones, which were obtained through endpoint dilution, showed that the frequency of the second-most abundant read was less than 0.64 % of the most abundant one. The resulting reads were aligned such that coding frames were maintained.

### Data analysis

Viral tropism of each sequence or read obtained using population-based sequencing and deep sequencing, respectively, was interpreted using G2P 2.5 [26] with a false-positive rate (FPR) cut-off of 10 % according the European guidelines [3]. Phylogenetic trees were constructed from the C2V3C3 sequences using the neighbor-joining method and the Maximum Composite Likelihood model and were assessed using the Bootstrap test (1,000 replicates) performed using MEGA version 6 software [27]. Putative dual infection was suggested according to previously proposed criteria: in the *env* C2V3 region, when the two clusters in the phylogenetic tree were separated by a branch with a bootstrap value >90 % obtained from more than 500 randomly selected reads, and when the net mean distance between the two groups was >5 % [28, 29]. Statistics was conducted using IBM SPSS Statistics version 21 software (IBM, Tokyo, Japan). The level of significance was set at 0.05.

Nucleotide sequences reported are available in the DDBJ databases with the accession numbers AB981480–AB981579 for population-based sequencing data and DRX021920–DRX021929 for deep sequencing data.

## Results

The characteristics of 50 treatment-naive patients (P1 through P50) included in the study are shown in Table 1. The majority of participants (88 %) were homosexual males. The age, CD4 count, and viral load varied widely among the patients. All the viral strains were typed as subtype B.

**Table 1:** Characteristics of the patients included in the study

Patients, *n*	50
Age (years), median (range)	36.5 (21–67)
Sex, *n* (%)	
Male	49 (98)
Female	1 (2)
Route of transmission, *n* (%)	
Homosexual	44 (88)
Heterosexual	6 (12)
HIV-1 subtype, *n* (%)	
Subtype B	50 (100)
CD4 (cells/mm^3^), median (range)	239.5 (8–596)
Viral RNA load (log_10_ copies/mL),	
median (range)	4.51 (3.00–6.32)
Proviral DNA load (log_10_ copies/10^6^ PBMCs),	
median (range)	2.94 (2.24–4.14)
Subjects with drug-resistant virus, *n*	18
To NRTI	6
To NNRTI	13
To PI	2

Population-based C2V3C3 sequencing was successfully performed using plasma RNA and PBMC DNA from the same blood samples in all the patients. The tropism of the viral RNA and proviral DNA V3-coding sequences was predicted using G2P with an FPR threshold of 10 %. X4 tropism was inferred in 11 samples using viral RNA and in 14 samples using proviral DNA. There was no statistically significant difference in CD4 counts between the subjects with R5 and those with X4 when either viral RNA or proviral DNA was used for tropism prediction (Table 2). Discordance in the tropism between the two compartments was seen in five subjects (10 %). Their characteristics are shown in Table 3. Four subjects (P10, P25, P27, and P45) showed R5 virus in the viral RNA and X4 virus in the proviral DNA, and one subject (P8) showed X4 virus in the viral RNA and R5 virus in the proviral DNA. CD4 counts of these subjects were 112 (P8), 390 (P10), 242 (P25), 419 (P27), and 113 (P45) cells/μl. The *env* V3 amino acid sequences of these five discordant samples are shown in Table 4. The numbers of different amino acids between the viral RNA and the proviral DNA were 1, 3, 5, 8, and 10 for P8, P27, P10, P25, and P45, respectively.

**Table 2:** Relationship between coreceptor Tropisms and CD4 Counts

Compartment	Coreceptor tropism	n	CD4 counts (mean ± SD)	P
Plasma RNA	R5	39	253 ± 152	0.38
	X4	11	201 ± 219	
PBMC DNA	R5	36	245 ± 154	0.83
	X4	14	233 ± 206

**Table 3:** Characteristics of the five patients whose virus showed discordant tropisms between plasma RNA and PBMC DNA

Patient	Age	Sex	Route of transmission	CD4 (cells/mm^3^)	Viral RNA load (log_10_ copies/mL)	Proviral DNA load (log_10_ copies/10^6^ PBMCs)	Major drug resistance mutations
P8	27	male	homosexual	112	5.23	3.19	None
P10	26	male	homosexual	390	4.80	3.54	L74F, V118I in RT
P25	55	male	homosexual	242	4.51	2.62	V179D in RT
P27	52	male	homosexual	419	3.76	2.61	M41L, M184V, T215Y, K103N, V108I in RT
P45	34	male	homosexual	113	5.51	2.66	None

**Table 4:** Amino acid sequences of the env V3-coding region of the samples with discordant coreceptor tropism predictions between plasma RNA and PBMC DNA

Patient	Compartment	V3 amino acid sequences																																			FPR (%)
P8	Plasma RNA	C	**A**	R	P	N	N	N	T	R	K	S	V	S	M	G	P	G	K	V	M	Y	A	T	G	A	I	I	G	D	I	R	Q	A	H	C	8.7
	PBMC DNA	C	**T/A**	R	P	N	N	N	T	R	K	S	V	S	M	G	P	G	K	V	M	Y	A	T	G	A	I	I	G	D	I	R	Q	A	H	C	14.3
P10	Plasma RNA	C	T	R	P	N	**N**	N	T	R	K	S	I	**H**	I	G	P	G	R	A	F	**Y**	A	T	G	**D**	I	T	G	D	I	R	K	A	**H**	C	40.1
	PBMC DNA	C	T	R	P	N	**S**	N	T	R	K	S	I	**R**	I	G	P	G	R	A	F	**V**	A	T	G	**G**	I	T	G	D	I	R	K	A	**Y**	C	1.7
P25	Plasma RNA	C	T	R	P	N	**N**	**N**	T	R	K	**S**	I	H	I	G	P	**G**	R	A	F	Y	A	T	**G**	**D**	I	I	**G**	**E/D**	I	R	Q	A	H	C	62.5
	PBMC DNA	C	T	R	P	N	**S**	**I**	T	R	K	**T**	I	H	I	G	P	**R**	R	A	F	Y	A	T	**R**	**Q/R**	I	I	**E**	**N**	I	R	Q	A	H	C	14.7
P27	Plasma RNA	C	T	R	P	N	N	N	T	R	K	G	**R**	H	M	G	P	G	G	A	F	W	A	**T**	G	**E**	I	I	G	N	I	R	Q	A	H	C	89.1
	PBMC DNA	C	T	R	P	N	N	N	T	R	K	G	**I**	H	M	G	P	G	G	A	F	W	A	**R**	G	**D**	I	I	G	N	I	R	Q	A	H	C	7.4
P45	Plasma RNA	C	T	R	P	N	**N**	N	T	I	**K**	**S**	I	**H**	**L**	G	P	G	**Q**	A	**L**	Y	T	T	**-**	**D**	I	**I**	G	D	I	R	Q	A	H	C	89.1
	PBMC DNA	C	T	R	P	N	**S**	N	T	I	**R**	**R**	I	**P**	**I**	G	P	G	**R**	A	**F**	Y	T	T	**G**	**R**	I	**-**	G	D	I	R	Q	A	H	C	1.1

Amino acids that differed between DNA and RNA samples from the same patient are indicated in bold

**Fig. 1:**
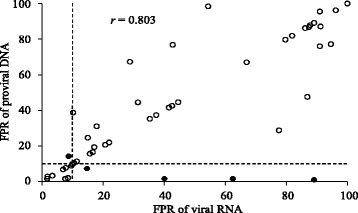
Scatter plot of FPR values in DNA sample versus those in RNA samples that were obtained using G2P 2.5 based on population-based sequencing. Viral tropism was interpreted using an FPR cut-off of 10 % shown as a dotted line. Tropism-concordant pairs are shown by open circles, and discordant pairs are shown by closed circles. A correlation coefficient was 0.803

**Fig. 2:**
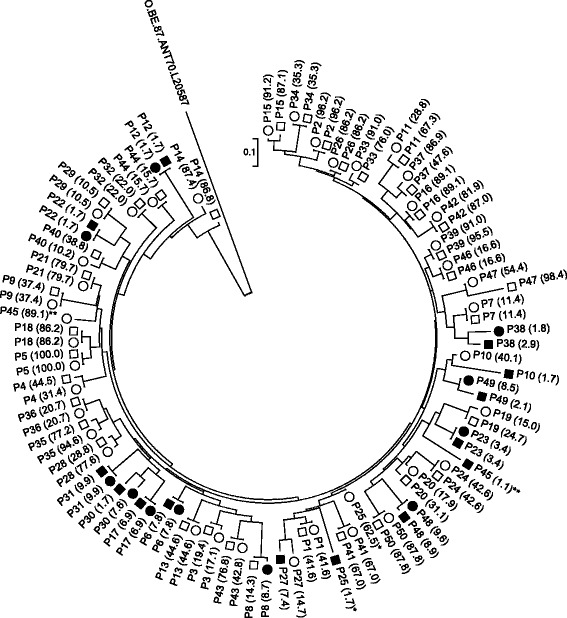
Phylogenetic tree constructed from population-based proviral DNA and viral RNA paired C2V3C3 nucleotide sequences of 50 subjects. R5 viruses in the RNA samples are shown by open circles, and R5 viruses in the DNA samples are shown by open squares; X4 viruses in the RNA samples are shown by filled circles, and X4 viruses in the DNA samples are shown by filled squares. The FPR for each sequence is shown in parentheses. Two discordant samples (P25 and P45) are indicated by asterisks. A Group O sequence (O.BE.87.ANT70.L20587) is used as an outlier

The FPRs in the two compartments are plotted in Fig. 1, yielding a correlation coefficient of 0.803. A complete agreement in the FPR between the two compartments was observed in 26 of the 50 samples. Thirteen samples had a higher FPR for the viral RNA, and 11 samples had a higher FPR for the proviral DNA. The FPRs of the viral RNA and the proviral DNA showed no statistically significant difference (*P* = 0.37 using a paired Student’s *t*-test). No relationship was found between the CD4 counts and the FPR (data not shown). A phylogenetic analysis of the C2V3C3 region, as shown in Fig. 2, revealed that paired samples of viral RNA and proviral DNA formed clusters of sequences with high bootstrap support, except for two cases (P25 and P45).

**Fig. 3:**
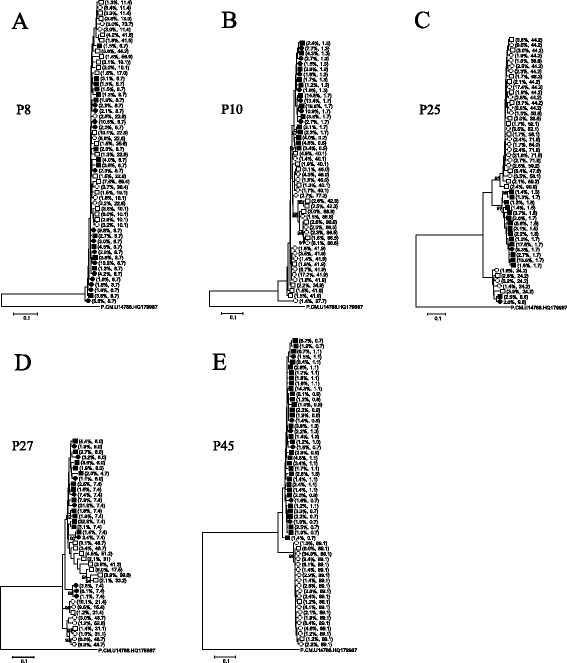
Phylogenetic trees constructed from proviral DNA and viral RNA C2V3C3 nucleotide sequences obtained from subjects P8 (**a**), P10 (**b**), P25 (**c**), P27 (**d**), and P45 (**e**) using deep sequencing. R5 viruses in the RNA samples are shown by open circles, and R5 viruses in the DNA samples are shown by open squares; X4 viruses in the RNA samples are shown by filled circles, and X4 viruses in the DNA samples are shown by filled squares. The frequency in the RNA or DNA compartment and the FPR for each sequence are shown in parentheses. A Group P sequence (P.CM06.U14788.HQ179987) is used as an outlier

To investigate the discrepancy in tropism between the viral RNA and the proviral DNA in the five discordant cases at a subpopulation level, we performed a deep sequencing analysis of the PCR products from the RNA and DNA samples. Phylogenetic trees generated from the deep sequencing data are shown in Fig. 3. In three cases (P8, P10, and P27) in which the population-based RNA and DNA sequences were located next to each other in the phylogenetic tree (Fig. 3), the RNA and DNA variants generated single clusters when examined using deep sequencing. The total frequency of the R5 variants was 51.7 % for the RNA sample and 63.1 % for the DNA sample for P8, 67.2 % for RNA and 36.3 % for DNA for P10, and 51.7 % for RNA and 41.0 % for DNA for P27. In the two cases (P25 and P45) in which the population-based RNA and DNA sequences were phylogenetically distant from each other (Fig. 3), variants from the RNA and DNA samples formed more than two distinct clusters, supported by bootstrap values of >90 % and net mean distance of >5 %. P25 had three clusters: two were dominated by R5-tropic variants, and one was dominated by X4-tropic variants. P45 exhibited two clusters: one was composed of R5-tropic variants, and the other was composed of X4-tropic variants. In both cases, the variants observed in the viral RNA occurred more frequently in R5-dominant clusters, while the variants observed in the proviral DNA occurred more frequently in X4-dominant clusters. Specifically, P25 had R5 variants at a frequency of 89 % and X4 variants at a frequency of 11 % in plasma, and R5 variants at 39 % and X4 variants at 61 % in PBMCs; P45 had R5 variants at 90 % and X4 variants at 10 % in plasma, and R5 variants at 8 % and X4 variants at 92 % in PBMCs. To confirm the presence of plural clusters in these two subjects, we repeated deep sequencing using the same plasma and PBMC samples, showing that P25 and P45 had three and two distinct clusters, respectively, which were characterized by different frequencies of R5 and X4 variants.

## Discussion

Tropism testing is required prior to the administration of a CCR5 antagonist for the treatment of HIV-1 infected individuals. Although clinical evaluations have been generally based on plasma-based genotypic assays, cell-based assays are favored for patients whose viral load is suppressed by ART but for whom a drug change is being considered because of adverse effects or regimen simplification [3]. Although both assays were predictive of a virologic response, discrepancies between genotypic tropism predictions using viral RNA and those using proviral DNA have been reported in many studies [16, 18–21, 23].

In this study, we analyzed the tropism of viral RNA and proviral DNA in 50 treatment-naive HIV-1 infected patients by population-based sequencing. The results showed tropism discordance between RNA and DNA in five patients. We further analyzed the viral RNA and proviral DNA sequences of these five cases by deep sequencing. The phylogenetic trees of three of them showed that both R5-tropic variants and X4-tropic variants assembled within the same cluster. The total frequencies of the R5 variants in these cases were moderately different between the RNA and DNA compartments, which almost agreed with the population-based sequencing results. In general, the emergence of X4-tropic variants is associated with disease progression [1]. However, the X4-tropic virus population dose not increase constantly, and its kinetics sometimes differs between plasma and replication-competent PBMCs [30, 31]. The tropism discrepancies observed using population-based sequencing in these three cases may have been caused by temporal fluctuations in the ratio of R5-tropic to X4-tropic variants in the plasma and PBMC compartments.

In the other two cases, phylogenetic analysis showed three clusters for P25 and two clusters for P45 that were supported by high bootstrap values. Each cluster was dominated by either R5-tropic variants or X4-tropic variants. In both cases, the total frequency of the R5 variants was higher than that of the X4 variants in the plasma compartment, and the opposite pattern was observed in the PBMC compartment. Taken together, these data indicate that the HIV-1 infection in the two cases was composed of two distinct strains: one strain was R5-tropic and plasma-oriented while the other strain was X4-tropic and cell-oriented. Coexistence of these distinct strains *in vivo* may have resulted from dual infection [28, 29], which is defined by either simultaneous or consecutive infections with different viral strains.

In general, X4-tropic sequences are more commonly predicted from proviral DNA than from viral RNA in population-based sequencing [3] and deep sequencing [17, 32, 33]. In cases P25 and P45 in this study, variants belonging to X4-predominant clusters occurred more frequently in DNA than in RNA. These findings suggest that X4 variants may be more likely than R5 variants to reside in cells as proviral DNA. The differential compartmentation of R5 and X4 viruses has not yet been fully explained. One possible explanation is that the R5 virus has a higher replication ability than the X4 virus, resulting in the production of more R5 virions in the plasma and the death of cells carrying R5 proviral DNA. The lower replication ability of the X4 virus may be caused by the accumulation of mutations. X4 variants have been shown to have evolved from R5 populations through progressive mutations in the *env* V3-coding region [34–36]. During this process, the other part of the viral genome may also suffer from mutations, resulting in the deterioration of the replication ability. On the other hand, the X4 virus has been referred to as a syncytium-inducing virus because of its ability to induce syncytia in the MT-2 cell line expressing X4. However, as Coakley et al. [30] discussed, this syncytium-forming ability of the X4 virus does not necessarily imply an enhanced cytopathogenicity. The R5 virus has been shown to be equally or more cytopathogenic to its host cells than the X4 virus [37, 38].

## Conclusions

In the present study, we showed that phylogenetically distinct clusters of HIV-1 variants, which may have been generated by dual infection, was involved in the discrepancies between HIV-1 tropism predictions from RNA and DNA. Although the number of patients with tropism discordance between RNA and DNA was small, out results suggest that the tropism testing using PBMC DNA or deep sequencing would have to be considered when the viral load is not suppressed or rebounds in the course of maraviroc-containing regimens, as previously proposed by Swenson et al. [32]. Further studies are needed to address the clinical relevance of tropism discordance in viral RNA and proviral DNA.

## Abbreviations

ART: Antiretroviral therapy
FPR: False-positive rate
G2P: Geno2pheno [co-receptor]
gp120: Envelope glycoprotein
MVC: Maraviroc
NNRTI: Non-nucleoside reverse transcriptase inhibitor
NRTI: Nucleoside reverse transcriptase inhibitor
PBMCs: Peripheral blood mononuclear cells
PI: Protease inhibitor
PTP: PicoTiterPlate
R5: CCR5-specific
R5X4: Using both CCR5 and CXCR4
X4: CXCR4-specific
